# Telephone-based motivational interviewing versus usual care in primary care to increase physical activity: a randomized pilot study

**DOI:** 10.1186/s40814-019-0390-0

**Published:** 2019-01-15

**Authors:** Deborah Rohm Young, Miki K. Nguyen, Ayae Yamamoto, Magdalena Pomichowski, Melissa Cornejo, Silvia Paz, Karen J. Coleman, Robert E. Sallis, Stephen P. Fortmann

**Affiliations:** 1 Department of Research & Evaluation, Kaiser Permanente Southern California, 100 S. Los Robles, 2nd Floor, Pasadena, CA 91101 USA; 2 Kaiser Permanente Southern California, 9961 Sierra Ave, Fontana, CA 92335 USA; 3 Kaiser Permanente Center for Health Research, 3800 N. Interstate Avenue, Portland, OR 97227 USA

**Keywords:** Diabetes, Prediabetes, Physical activity, Primary care settings

## Abstract

**Background:**

Diabetes and prediabetes are chronic conditions that affect over 40% of the US adult population combined. Regular physical activity can benefit people with diabetes through improved glucose control and can reduce the conversion of prediabetes to diabetes. Studies are needed in settings where people with these conditions can be identified and provided the skills and support to increase physical activity. The primary care setting meets this need, but there are insufficient high-quality trials to recommend this approach be broadly implemented.

**Methods:**

We conducted a randomized, 24-week pilot study in Southern California to assess the feasibility of using information technology systems available in primary care for identifying potential participants, test methods for obtaining physical activity clearance, conducting mail-based assessments, and delivering telephone-based motivational interviewing to increase physical activity. Eligibility criteria included age between 18 and 74 years, diabetes or prediabetes, and physically inactive based on a clinical assessment tool. At baseline and follow-up, physical activity was assessed by a 7-day accelerometry, cardiometabolic risk factors were collected from electronic medical records, and psychosocial factors were assessed from validated questionnaires administered through a mail survey. Participants were block randomized into intervention or usual care. Staff collecting outcome data were blinded to group assignment. Analysis of covariance was used to assess the difference at follow-up between the intervention and usual care, adjusting for baseline.

**Results:**

A total of 67 participants were randomized. Follow-up mail assessments were completed by 53 participants. Of 224 potential intervention calls, 194 were completed (87%). Psychosocial measures significantly improved in four of the five factors for physical activity motivation relative to participants in the usual care arm. The more internally focused factors for exercise self-regulation and outcome expectancies scores were significantly greater for participants in intervention compared with usual care. Moderate to vigorous physical activity improved in intervention participants relative to usual care, but the difference was not statistically significant. No adverse events were noted.

**Conclusions:**

The objectives of this pilot study were met. If a fully powered trial is successful, primary care settings with “behind-the-scenes” information technology support may be appropriate to increase physical activity among patients with prediabetes and diabetes.

**Trial registration:**

Exercise Promotion in Primary Care (EPPC), NCT03429088, registered on February 5, 2018.

## Background

Diabetes is a chronic disease that is increasing in prevalence. About 30.3 million people have diabetes—9.4% of the US population [1]. Another 84 million adults, or about ½ of the US population, have prediabetes, a condition of abnormally high blood glucose levels and a precursor to diabetes [1].

Regular physical activity markedly improves health for those with diabetes and prediabetes. For patients with diabetes, regular physical activity training significantly improves glucose control [2] and can induce remission [3]. Intense lifestyle interventions, including physical activity and weight loss, can reduce the transition of prediabetes to diabetes [4]. However, this knowledge is insufficient to motivate Americans to increase their physical activity. Based on accelerometry data from the National Health and Nutrition Examination Survey (NHANES), 34% are extremely inactive (accumulate about 5 min of moderate to vigorous physical activity (MVPA) per day) [5] and only 5% meet national guidelines of 150 min per week [6, 7].

The health care sector is an important setting to promote physical activity because of its ability to reach and influence large numbers of people with diabetes and prediabetes. Brief health care provider physical activity assessment and counseling can successfully increase physical activity in the short-term (< 6 months) [8–11], but advice during an outpatient visit is insufficient for lasting improvements. The US Preventive Services Task Force reported limited information on physical activity counseling interventions on cardiometabolic risk factors, that well-conducted trials are needed to increase physical activity, and called for more studies of such interventions in primary care settings that require minimal health care resources [12]. Telephone-based counseling for physical activity is evidence-based [13], and although healthcare systems routinely use this technology for care management, it has not been studied for physical activity promotion in this setting. Additionally, objective measures to assess physical activity are needed [14].

In response to these recommendations, we conducted a pilot study to determine if telephone counseling, in a health care setting that routinely assesses the physical activity of its patients, is a feasible approach to improve physical activity among patients with diabetes and prediabetes. We built on an existing innovation at Kaiser Permanente, in which physical activity is assessed at every outpatient visit and recorded in their electronic medical record (EMR) using a “Exercise Vital Sign” (EVS). The primary aims were to pilot study elements, including identification of eligible patients; health care provider recruitment, training, and engagement; patient recruitment; data collection procedures; and a 24-week intervention.

## Methods

### Study design

This pilot tested the elements of a two-armed study with parallel group of a 24-week physical activity intervention among physically inactive prediabetic and diabetic patients not prescribed insulin. A sample size of 60 was chosen to provide a sample large enough to test study procedures and allow for a range of intervention experiences by participants. After completion of baseline data collection, participants were randomized to intervention or usual care conditions. The intervention consisted of telephone counseling, using motivational interviewing (MI) [15] and individualized support, to assist participants in increasing and maintaining their physical activity. We assessed physical activity assessed from accelerometers, psychosocial measures such as quality of life, weight change, and cardiometabolic risk factors such as blood pressure and glycated hemoglobin (HbA1c). All measures were assessed at baseline and following the 24-week intervention. Accelerometers and surveys were mailed to participants and returned in previously addressed, stamped envelopes. Weight and cardiometabolic risk factors were extracted directly from the EMR. After study completion, selected participants, clinic staff, and providers were recruited for one-in-one interviews regarding feasibility and acceptability of the study design and methods. The study was approved by the Kaiser Permanente Southern California Institutional Review Board, IRB # 10106.

### Participant eligibility

The target population was physically inactive individuals (i.e., less than 30 min of physical activity per week as assessed by the EVS) between age 18 and 74 years, with a primary care visit in the previous 2 years, having either prediabetes or diabetes without insulin and able to communicate in English. All patients had to be cleared by their primary care provider to increase their physical activity. Prediabetes was defined as a HbA1c level between 5.7 and 6.4%, a fasting blood glucose level between 100 and 125 mg/dL, a 2-h oral glucose tolerance test between 140 and 199 mg/dL, or at least one outpatient ICD-9 or ICD-10 diagnosis code for abnormal glucose levels, all within the past 6 months. Diabetes was defined as having at least two outpatient ICD-9 or ICD-10 diabetes diagnoses in the EMR in the past 3 years, and one of the following: a HbA1c value at or greater than 6.5% in the past 2 years or a fasting blood glucose greater than 125 mg/dL in the past 2 years. Exclusion criteria were health conditions that might limit individuals from participating in physical activity without professional supervision, including obesity class 3 (body mass index (BMI; kg/m^2^ > 40 kg/m^2^), disabling rheumatoid or osteoarthritis, and ongoing treatment for cancer or unstable coronary heart disease.

Physical activity was assessed at each outpatient visit by trained medical assistants who asked patients two questions: “On average, how many days per week do you engage in moderate to strenuous exercise (like a brisk walk)?” and “On average, how many minutes do you engage in exercise at this level?” Response choices for days were 0–7, and minutes were recorded as 0, 10, 20, 30, 40, 50, 60, 90, 120, and 150 min or greater. The responses were recorded in each patient’s EMR, and the associated software calculated minutes per week of MVPA. Face and discriminant validity of the EVS are adequate [16], and being physically active (EVS > 150 min per week, compared to 0 EVS minutes per week) was associated with lower HbA1c values [17].

### Clinic recruitment

Study staff identified primary care office clinics located in Southern California that might be interested in participating in the pilot study. After initial interest, staff met with clinic leadership to explain study goals, further gauge interest, and identify methods to recruit patients. Because of the pilot’s small scope, it was determined that pre-eligibility would be assessed by study staff and primary care providers would assess potentially eligible patients for their ability to increase physical activity and provide clearance through the EMR. Methods to remind providers to clear patients were identified, and tools were created. These included (1) 4 × 6 in. cards of eligibility criteria with contact information if patient was eligible that could be placed in the exam room when rooming a potentially eligible patient, (2) a bright yellow card indicating the patient should be assessed for eligibility, and (3) creating an automatic note in the EMR that would auto-populate stating whether a patient was or was not cleared for exercise.

Study staff attended a monthly primary care provider and clinic staff meeting to present the study’s background and rationale and explain the “ask;” that is, to determine if a potentially eligible patient could safely increase his/her physical activity, and if so, to clear them for exercise by either placing a code in the patient’s EMR or asking the clinic staff to relay the information to study staff. Neither providers nor clinic staff were asked to recruit participants.

### Participant recruitment

A program code run against the EMR database was used to determine possible eligibility according to the study inclusion and exclusion criteria. The code was run twice weekly to identify potentially eligible patients who had clinic appointments that week. Initially, lists were sent to clinic staff and brightly colored notecards were placed in examination rooms to identify patients to be assessed for possible medical contraindications for increased physical activity. The evaluation was based on general knowledge of the patients’ medical conditions. Patients cleared for physical activity were called by study staff, the study purpose was explained, eligibility was confirmed, and if interested, they were invited to participate in the pilot. Informed consent was mailed to potential participants, and after written consent was obtained, participants underwent baseline data collection.

Many potentially eligible patients were not assessed for their ability to increase physical activity during their medical clinic visit because the primary care provider forgot to make the assessment. A revised plan was enacted in which study staff directly reached out to potentially eligible participants through mail and follow-up telephone calls. If interested, study staff directly contacted the corresponding provider to ascertain if it was safe for the patient to increase physical activity. Once a patient was cleared for increased physical activity, the same procedures were followed to obtain written informed consent.

### Baseline and follow-up assessments

#### Moderate to vigorous physical activity

Physical activity was assessed with Actigraph® model 7185 accelerometers. After contacting the participant to confirm timing, monitors were mailed to participants via 1-day return receipt mail along with printed instructions on its use. Accelerometers were initialized prior to mailing and set to begin collecting data at 5 am on the day after the participants received the monitor. Participants were asked to wear a monitor during all waking hours for 7 consecutive days. Participants returned the monitors in envelopes provided by the study. The following count thresholds were used to assign each 60-s interval to a physical activity intensity category: sedentary (< 100), light (100–1951), moderate (1952–5723), and vigorous (> 5723 counts) [18]. At least 10 h per day of monitor wear for at least 4 days was required for a participant’s data to be used for analysis.

#### Cardiometabolic risk factors

We assessed BMI, HbA1c, blood pressure, and lipids at baseline and follow-up from the EMR. Baseline data were identified as the most recent prior to randomization. Follow-up was defined as 24 weeks after randomization. Data from office and laboratory visits closest to the follow-up accelerometry date were used; at times this required using some follow-up data from before 24 weeks.

#### Psychosocial measures

A survey was mailed to participants along with the accelerometer to complete and return. Quality of life was assessed by the SF-36 [19]. This widely adopted generic measure of 36 items yields scales of functional health and well-being as well as summary physical health and mental health scores. Reliability typically exceeds 0.90 [20]. Additional instruments were administered to assess constructs relevant to the intervention theory (described below). Perceived competence for participating in physical activity was assessed by the Exercise Self-Efficacy Scale [21] (12 items, *α* = 0.83–0.85). The scale has two factors: making time for exercise and sticking to it. Motivation for physical activity was assessed using the Motivation for Physical Activities Measure [22]. It is a 30-item scale with internal consistency greater than 0.87 in which participants are asked to indicate on a 5-point Likert scale their motivations for participating in physical activity, with factors of interest/enjoyment, competence, appearance, fitness, and social motivations. Physical activity self-regulation is a 16-item scale with response options ranging from 1 to 7, anchored with “not at all true” (1) and “very true” (7). The scale results in four factors: external regulation (participating in physical activity to gain an external reward or avoid punishment), introjected regulation (participating because of feelings of obligation or guilt), identified regulation (participating because of the value of the consequences), and intrinsic regulation (participating because it is consonant with one’s values) [23]. Relative Autonomy Index is formed from the factors, with higher scores indicating greater autonomy toward physical activity. The outcome expectations for exercise scale is a 9-item scale with 5-point Likert response options [24]. Internal consistency is 0.89. Social support for physical activity is a 13-item scale that queries separately for family and friends. Internal consistency ranges from 0.61–0.9) [25]. Two factors result for family and friends, “participation” and “rewards.” These instruments predict physical activity behavior change [26, 27].

Staff collecting outcome data were blinded to participant treatment assignment.

### Intervention

The conceptual model for the intervention was based on Self-Determination Theory (SDT) [28] which posits that motivation for a behavior ranges on a continuum from external to internal motivation. The behaviors that originate from one’s self (i.e., autonomous or internalized) are more likely to be maintained compared with behaviors resulting from external forces (which in the extreme can be coercive). When a person becomes autonomously motivated toward a health behavior, that behavior receives more effort, engagement, persistence, and is thus more likely to continue [28]. Autonomous self-regulation can be promoted by autonomy supportive social environments in which the individual is accepted, his/her frame of reference is acknowledged, information and encouragement is provided about making behavior change, and choices are respected [29]. SDT identifies 3 psychological needs that are critical to supporting intrinsic self-regulation: the need for autonomy, competency to achieve desired outcomes (i.e., self-efficacy), and relatedness to others.

We chose to MI as the tool to deliver the intervention, a technique successfully implemented health behavior change studies [30]. MI is based on the clinical principles of (1) expressing empathy, understanding, and acceptance, including understanding that ambivalence is normal; (2) identifying discrepancies between current behavior and important goals and facilitating the participant to present rationale for change, rather than using direct persuasion techniques; (3) allowing the participant to be resistant to change, so resistance can be explored and the participant can resolve resistance; and (4) supporting self-efficacy for change by identifying the participant’s strengths, encouraging his/her ability for change, and affirming successes and achievements [30, 31]. While SDT and MI were developed independently, there are considerable similarities [29, 32] and MI is a common approach for implementing SDT interventions [29, 32].

The intervention goal was to meet national physical activity guidelines of 150 min of MVPA per week [7]. The intervention was administered through behavioral counseling using MI by telephone. The interventionist, based in the research department, was a research associate with a master’s degree and broad knowledge in public health and health behavior change. She underwent a 30-h web-based MI program with webinars, skill-building activities and a 2-day face-to-face training with a MI expert trainer. A manual of procedures was created to prompt MI skills and guide physical activity-related information to share with participants during the calls. MI calls were scheduled at weeks 1, 2, 4, 6, 12, 16, and 24. Intervention participants received a packet that included information about target heart rate, rate of perceived exertion scale, expected changes while exercising (e.g., increased heart rate and breathing, perspiration), tips about exercising in hot and inclement weather, tips for motivation and reducing barriers, a 24-week physical activity log, and a KP-branded physical activity personal action plan.

The first MI call included introductions, intervention format and expectations, and information describing types of moderate intensity physical activity; verification that the participant knew how to monitor physical exertion through counting pulse rate or using the rate of perceived exertion scale; appropriate clothing and footwear for physical activity; and warm-up, cool-down, and flexibility exercises. The final MI call included a discussion on long-term maintenance strategies, plans to overcome boredom with an activity routine, and intrinsic and extrinsic rewards when goals are met.

Participants received approximately 2.5 h of telephone counseling, although for those who needed more support, more counseling occurred, and vice versa for those who needed less support. Because of the highly interactive nature of the motivational interviewing process, the intervention was not prescriptive, but rather designed to meet the participant where s/he was to increase physical activity and was empathetic but designed to create discrepancy between the participant’s current state and desired goals so change could be produced. Participants self-selected their physical activity intensity goal, which was typically at least of a moderate intensity (e.g., brisk walk) [33]. Weekly study staff meetings included case discussions on participants’ achievements and challenges toward goals; any medical-related issues participants had (e.g., hbA1c monitoring) were referred back to their primary care provider. The interventionist documented encounters and goals attained through progress notes in the EMR so the providers could monitor patients’ progress, if desired. After the last MI call, participants were mailed an action plan that was negotiated during the last call.

### Usual care

Participants randomized to the usual care arm received a 3-page physical activity resource handout that included fitness classes and facilities, their costs, and available walking paths located in their communities as well as online resources available through Kaiser Permanente.

### Qualitative assessment

After completion of the study, semi-structured interview guides were prepared for primary care providers, clinic staff, and participants to identify strengths and weaknesses of the study approach. Providers and staff who engaged in the study were asked about their familiarity with how patients were identified to be cleared for exercise, what the intervention components were, how they felt the intervention benefitted patients, and what challenges they faced with participating in the study. Participants were asked about potential measurement burden, their perceptions of the telephone format, what challenges they faced during the study, benefits they obtained, and recommendations for improving the intervention. Interview participants were those who had been assigned to the intervention arm and selected by their engagement in the intervention; those who completed most MI calls and those who missed a number were asked to participate to gain a wide range of opinions. Interviews were conducted in person (physicians and clinic staff) and by phone (participants), and all were audio recorded.

### Analysis

Participants were randomized by study staff into intervention vs usual care using a block randomization using blocks of 8, 10, and 12, stratified by sex. Study staff informed participants of their study assignment. Baseline characteristics of the intervention and usual care groups were compared using chi-square test for categorical variables and Wilcoxon rank sum test for continuous variables as appropriate.

An analysis of covariance was used to assess the difference in means in physiological, accelerometry, and survey response measures at 6 months of follow-up between the intervention and usual care, adjusting for their respective baseline measures. All analyses were performed with SAS Enterprise Guide 5.1. All tests of significance were exploratory. No adjustment of the overall level of significance for multiple testing was done.

Recordings of qualitative interviews were listened to verbatim by researchers and staff. Main themes were independently identified, summarized, and compared. When discrepancies were noted, results were compared, and consensus was obtained. Due to cost considerations, written transcripts were not prepared or analyzed.

## Results

### Recruitment

**Fig. 1:**
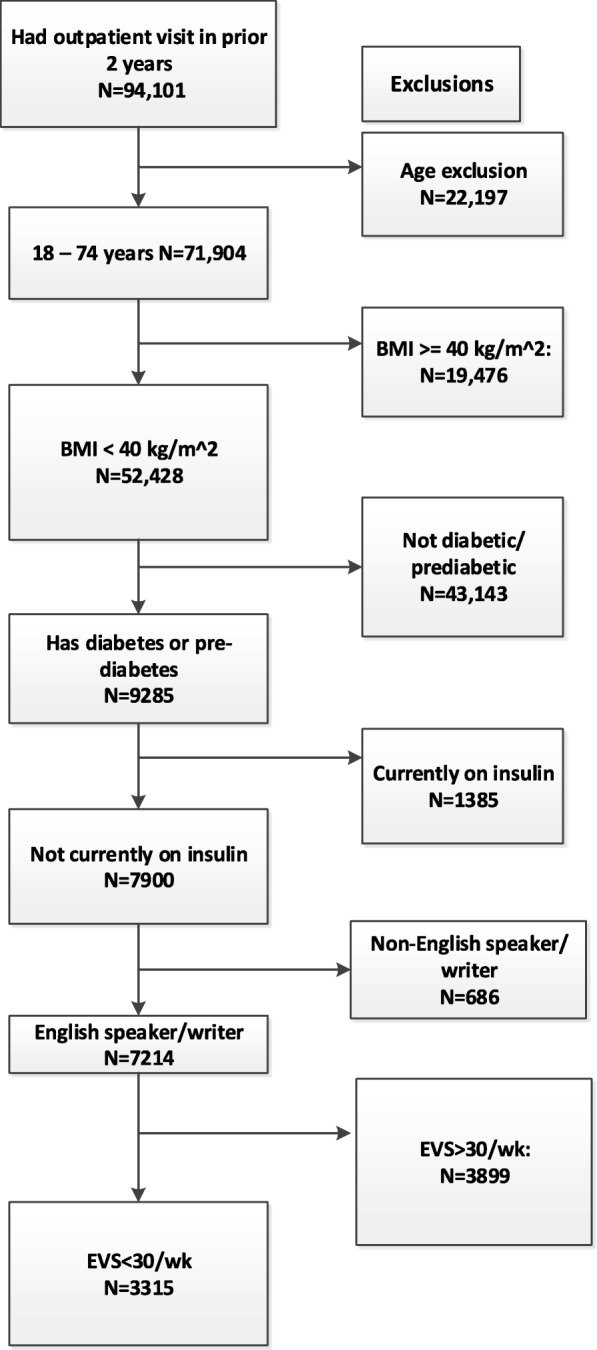
Sample flow chart

Figure 1 displays the participant recruitment process for clinic A (diabetic and prediabetic patients). From a potential pool of 94,101 patients who had an outpatient visit in the prior 2 years, 3315 met the basic eligibility criteria. Clinic B (diabetic patients only) had 637 diabetic patients that met eligibility criteria. Of the patients who were potentially eligible from both clinics, 504 had outpatient visits scheduled during the study’s recruitment period and were thus available to be recruited.

**Fig. 2:**
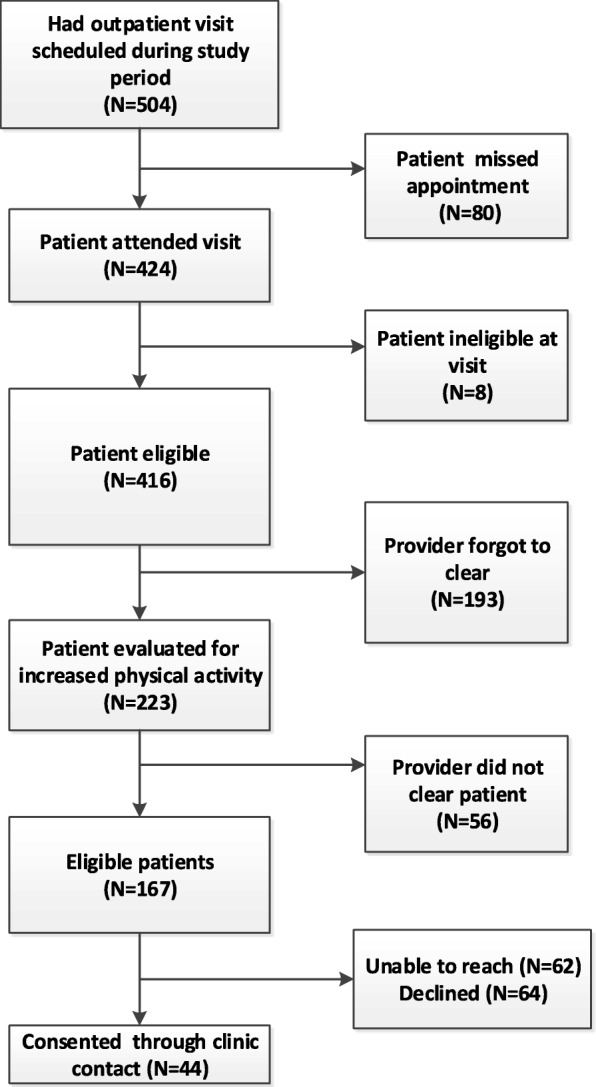
Recruitment flow chart

Figure 2 displays the clinic recruitment process. Recruitment began in November 2015 and continued through May 2016. Of the potentially eligible patients, 80 either did not attend or canceled their appointment, 8 were deemed to not meet eligibility criteria during the visit, and providers determined that it was not safe for 56 to increase their physical activity. Providers forgot to screen 46% of eligible patients who had a clinic visit, leaving 167 patients for study staff to be contacted and informed about the study. Of those, 38% actively declined and 37% were not able to be reached by phone, leaving 44 patients who consented to participate. The clinic recruitment methods took 35 weeks (i.e., 1.25 participants per week).

Directly mailing potentially eligible participants from both clinics took 11 weeks to recruit the remaining 23 participants. During this time, 330 letters were mailed explaining the study and informing patients they would receive a follow-up telephone call. Three letters were returned because of bad addresses, we were not able to reach 159 (48%) patients, and 117 (35%) declined, leaving 51 patients interested in the study. There were 8 interested patients who did not receive clearance to increase their physical activity, another 8 patients whose provider did not respond to us, and 12 patients who lost interest in the study. Thus, direct mail resulted in consenting 23 participants.

A total of 32 participants were randomized to the intervention arm; the remaining 35 were randomized to the control arm (Table 1).

**Table 1:** Participant yield by recruitment method

	Recruitment method	
	Clinic-based (35 weeks)	Mailing plus telephone follow-up (11 weeks)
Total screened	504	330
Provider cleared for exercise	167	35
Provider did not clear	56	8
Provider forgot	194	7
Patient canceled or missed appointment	88	
Patient declined participation	61(37%)	117 (35%)
Patient not able to be reached by telephone or mail	65 (39%)	162 (49%)
Patient consented	44 (26%)	23 (7%)

Data are expressed in *N* (%)

### Intervention delivery

The intervention was designed for each participant to receive 7 MI calls. Of 224 potential calls with 32 intervention participants, 194 were completed (87%); 5 participants dropped out of the intervention (16%). MI calls varied from 5 to 25 min.

### Baseline and follow-up assessments

Baseline descriptive statistics for intervention and control participants are presented in Table 2. Participants were 61% female, 33% Hispanic, 12% non-Hispanic black, 7% Asian/Pacific Islander, 40% non-Hispanic white, and 8% other. Their mean age was 61.2 (7.61) years, and 69% were obese. Most participants (60%) were either high school graduates or had attended college but not graduated. Consistent with eligibility criteria, 73.1% had 0 min of physical activity, determined from the EVS (data not shown). Because one clinic agreed to include only diabetic patients, almost ½ of participants had HbA1c levels above 6.4%. Baseline LDL-cholesterol and HDL-cholesterol mean values were normal. Baseline mean blood pressure met the current criteria for stage 1 hypertension. Accelerometry-based moderate-to-vigorous activity at baseline was 17.7 (14.53) minutes/day, with baseline values higher for the usual care group. There were no other differences between intervention and usual care participants at baseline.

**Table 2:** Baseline demographic, health, and moderate-to-vigorous physical activity characteristics

Variable	Total (*N* = 67)	Usual care (*N* = 35)	Intervention (*N* = 32)
Sex *n* (%) female	41 (61.2%)	21 (60%)	20 (62.5%)
Age (years)	61.2 (7.61)	61.6 (7.52)	60.8 (7.81)
Race			
White	27 (40.3%)	14 (40%)	13 (40.6%)
Hispanic	22 (32.8%)	13 (37.1%)	9 (28.1%)
Black	8 (11.9%)	5 (14.3%)	3 (9.4%)
Asian/PI	5 (7.5%)	0 (0%)	5 (15.6%)
Other	5 (7.5%)	3 (8.6%)	2 (6.3%)
Education category			
Less than high school	5 (7.5%)	2 (5.7%)	3 (9.4%)
High school graduate/some college	40 (59.7%)	20 (57.1%)	20 (62.5%)
College graduate or higher	22 (32.8%)	13 (37.1%)	9 (28.1%)
Body mass index (kg/m^2^)	31.9 (4.48)	32.5 (4.46)	31.2 (4.47)
Body mass index category			
Normal weight	4 (6%)	2 (5.7%)	2 (6.3%)
Overweight	17 (25.4%)	6 (17.1%)	11 (34.4%)
Obese	46 (68.7%)	27 (77.1%)	19 (59.4%)
Hemoglobin A1c (%)	6.8 (1.21)	6.9 (1.26)	6.8 (1.18)
Hemoglobin A1c category			
< 5.7%	6 (9%)	3 (8.6%)	3 (9.4%)
5.7–6.4%	26 (38.8%)	14 (40%)	12 (37.5%)
6.5–7.0%	15 (22.4%)	8 (22.9%)	7 (21.9%)
7.0–7.9%	8 (11.9%)	2 (5.7%)	6 (18.8%)
8.0–8.9%	7 (10.4%)	5 (14.3%)	2 (6.3%)
> 9.0%	5 (7.5%)	3 (8.6%)	2 (6.3%)
Systolic blood pressure (mmHg)	130.7 (15.53)	131.6 (15.38)	129.7 (15.88)
Diastolic blood pressure (mmHg)	75.4 (7.40)	76.3 (6.57)	74.3 (8.20)
Total cholesterol (mg/dL)	172.7 (36.20)	166.6 (31.39)	179.3 (40.28)
High-density lipoprotein cholesterol (mg/dL)	46.8 (12.33)	45.1 (11.34)	48.7 (13.24)
Low-density lipoprotein cholesterol (mg/dL)	97.4 (31.97)	91.7 (27.52)	103.6 (35.61)
Moderate-to-vigorous activity (min/day)	18.0 (15.13)	22.6 (17.97)	13.0 (9.11)
Median	15.2	16.1	12.9
Q1, Q3	8.0, 22.6	9.6, 29.3	3.5, 20.7
Range	(0.2–80.9)	(1.9–80.9)	(0.2–37.6)

Data are expressed in *N* (%) for categorical variables and means (SD) for continuous variables

A total of 53 participants (79%) completed the mail-based follow-up assessments, which were completed in December 2016. Of those randomized, we were unable to contact 5 participants, 3 returned the package with no data, 2 declined further participation, and 4 did not return the package. There were 46 participants (69% of randomized participants; 87% of those who had follow-up assessments) with sufficient accelerometer data for analysis. Reasons for non-analysis were accelerometer malfunction (*n* = 4) and inadequate wear time (*n* = 3).

MVPA increased non-significantly among participants in the intervention group and decreased in participants in the usual care group, with a mean baseline-adjusted difference of 7.41 daily minutes (95% CI −2.9, 17.4) (baseline adjusted 6-month MVPA min/day intervention, 24.8 [95% CI 16.6, 33.0]; usual care, 16.4 [95% CI 7.7, 25.2]) (Table 4). This is an average increase of more than 50 min per week.

Follow-up BMI and blood pressure data were available for 54 (80%) participants; HbA1c was available for only 46 (57%) participants. Because lipid values are periodically assessed in clinical care, we had 6-month follow-up values in the EMR for only 23 (34%) participants. There were no between-group differences in these measures (Table 3).

**Table 3:** Follow-up body mass index, blood pressure, and moderate-to-vigorous physical activity and 95% confidence intervals

	Usual care	Intervention	Adjusted mean difference
Body mass index (kg/m^2^)	*N* = 28 31.6 (30.6, 32.5)	*N* = 27 31.7 (30.8, 32.6)	0.15 (−1.16, 1.47)
Systolic blood pressure (mmHg)	*N* = 28 136.4 (129.7, 143.0)	*N* = 26 133.2 (126.3, 140.1)	−3.17 (−12.77, 6.43)
Diastolic blood pressure (mmHg)	*N* = 28 76.4 (72.1, 80.7)	*N* = 26 76.2 (71.8, 80.7)	−0.22 (−6.41, 5.98)
Moderate to vigorous activity (min/day)	*N* = 25 16.4 (7.7, 25.2)	*N* = 21 24.8 (16.6, 33.0)	7.41 (−2.9, 17.4)

Data are expressed in adjusted mean (95% confidence intervals). Means are adjusted for baseline values

Health-related quality of life did not differ between participants in the intervention versus the usual care arm. There was a significant improvement for participants in the intervention compared with those in usual care for four of the five factors for physical activity motivation, with the social motivation factor approaching significance (baseline-adjusted mean difference 0.60 [95% CI 0.00, 1.20]) (Table 4). The more internally focused factors for exercise self-regulation were significantly greater for participants in intervention compared with those in usual care, as were outcome expectancies scores (baseline adjusted mean difference 0.28 [95% CI 0.02, 0.54]). Physical activity self-efficacy and social support scores did not differ for participants in the intervention compared with participants in the usual care arm.

**Table 4:** Follow-up psychosocial assessments and 95% confidence intervals and mean group differences

	Usual care	Intervention	Adjusted mean difference
SF-36			
Physical health composite t-score	45.73 (41.73, 49.74)	46.22 (42.47, 49.97)	0.48 (− 4.49, 5.45)
Mental health composite t-score	51.50 (46.24, 56.75)	47.50 (42.36, 52.64)	− 3.99 (− 10.59, 2.61)
General health composite t-score	48.65 (43.81, 53.48)	46.15 (41.39, 50.92)	− 2.49 (− 8,63, 3.64)
Motivations for physical activity score			
Enjoyment	3.50 (3.11, 3.89)	4.14 (3.75, 4.54)	0.64 (0.09, 1.20)
Competence	3.72 (3.31, 4.13)	4.36 (3.94, 4.87)	0.64 (0.05, 1.23)
Appearance	4.16 (3.76, 4.57)	4.85 (4.44, 5.27)	0.69 (0.11, 1.27)
Fitness	5.49 (5.11, 5.88)	6.29 (5.90, 6.68)	0.79 (0.25, 1.34)
Social	2.48 (2.06, 2.89)	3.08 (2.65, 3.50)	0.60 (0.00, 1.20)
Exercise self-regulation score			
External regulation	1.24 (1.05, 1.43)	1.23 (1.04, 1.43)	− 0.01 (− 0.28, 0.26)
Introjected regulation	2.37 (1.96, 2.77)	2.68 (2.26, 3.10)	0.31 (− 0.28, 0.26)
Identified regulation	5.20 (4.77, 5.64)	5.93 (5.48, 6.38)	0.73 (0.11, 1.36)
Intrinsic regulation	3.96 (3.53, 4.38)	4.66 (4.22, 5.10)	0.71 (0.10, 1.32)
Relative autonomy index	10.58 (9.33, 11.83)	12.85 (11.55, 14.15)	2.27 (0.46, 4.07)
Outcome expectancies score	4.02 (3.84, 4.20)	4.30 (4.11, 4.49)	0.28 (0.02, 0.54)
Physical activity self-efficacy score			
Making time to exercise	3.71 (3.28, 4.13)	3.77 (3.36, 4.17)	0.06 (− 0.47, 0.59)
Sticking to exercise	4.04 (3.68, 4.40)	4.04 (3.68, 4.40)	0.00 (− 0.46, 0.47)
Social support—family score			
Participation	19.78 (16.43, 23.13)	19.18 (15.92, 22.45)	− 0.60 (− 4.92, 3.72)
Rewards	3.47 (2.45, 4.49)	3.28 (2.27, 4.29)	− 0.19 (− 1.51, 1.13)
Social support—friends score			
Participation	15.89 (12.75, 19.03)	15.11 (12.17, 18.06)	− 0.77 (− 4.71, 3.16)
Rewards	3.46 (2.53, 4.38)	2.81 (1.93, 3.68)	− 0.65 (− 1.82, 0.52)

Data are expressed in adjusted means (95% confidence intervals). Means are adjusted for baseline values

No study-related adverse events were noted for either treatment arm.

### Intervention acceptance

Each of study participants interviewed (*n* = 4) (all intervention) was highly favorable on all aspects of the study design and implementation (Table 5). They stated that the calls helped with accountability, provided support and encouragement, and educated them about increasing their physical activity. They also mentioned that it would be nice to meet the interventionist in person to enhance the personal interaction. Health care providers (*n* = 7) and clinic staff (*n* = 4) thought the study was an excellent idea, that it was something that all their patients could benefit from, had minimal effect on their workflow, and that if it helps their patients, it is worth the extra time (Table 5). Even with the tools provided to remind providers to assess their patient’s ability to increase physical activity, they often forgot to make the assessment. They stated that clearing patients for exercise would be easier if it was part of their routine and offered suggestions of incorporating it into existing reminders imbedded into the EMR. They also suggested that providing staff support at the clinic would be a helpful reminder.

**Table 5:** Interview questions and responses from primary care providers (*n* = 4), clinic staff (*n* = 6), and participants (*n* = 4)

Sample questions of interview domain	Sample responses
Feasibility and acceptance (provider/staff)	
In what ways did the training/presentation support or not support you in understanding the pilot? What did you like about the pilot? What did you not like? What is your familiarity of the criteria for clearing/not clearing patients for physical activity?	Providers: Training was clear. Understood the criteria. Excellent idea to offer this resource to patients. On-site support person would be helpful. Hard to remember this option—needs lots of reminders. Integrate into proactive office encounter to make it easier to remember. Staff: Did not impact workflow. If we knew more about the study we could have encouraged the patients to exercise. Integrate patient clearance into the electronic medical records.
Intervention benefits/challenges to patients (provider/participants)	
What benefits do you see this pilot having on your patients (you) and their health outcomes? What do you view as the major challenges your patients (you) face in managing their diabetes or prediabetes? How do you think this intervention may have helped them (you)?	Providers: Exercise is as good as any medicine I can give them. Long-term, it may help them lost weight and manage their A1c. Hard to engage patients who are not motivated to increase physical activity or manage their health. Participants: A1c much better because of physical activity. I feel healthier than I have for a while.
Barriers/future opportunities (provider/staff)	
Is the pilot helpful to your practice? Has it been a hindrance or burden in any way? How would you feel if the pilot is adopted and integrated into our care delivery?	Providers: The extra steps were minimal and not burdensome. Let us identify additional patients who may benefit from exercise. Staff: Need to know why we are doing the study. Older patients may not be able to do physical activity.
Study aspects (participants)	
What is your feedback on the frequency of the phone calls, the length of the study, the telephone intervention, the study instruments, the accelerometer?	Participants: Easy to take calls. Not a time burden. Survey length is good. Wearing the accelerometer was fine.
Telephone physical activity counseling (participants)	
What resources were available to you to help you increase and maintain your physical activity? Was the intervention helpful? Was it a hindrance or burden? How does the phone counseling fit with your daily life activities? How can the program be more effective?	Participants: Found a place to walk that is safe. Felt supported and helped me make changes. Helped with accountability. In-person visit to meet counselor would be nice. May want to consider email/texts if cannot make calls.

## Discussion

Results from this pilot study suggest that it is feasible to identify potential eligible diabetic and prediabetic participants from EMRs, have their ability to increase physical activity assessed in primary care, data collection conducted through the mail, be randomized, administer an intervention entirely by telephone, and obtain follow-up data through mail and EMRs. Participants viewed the intervention positively, and primary care providers and clinic staff reported the study was an excellent idea and one that can benefit patients. Results indicate that intervention participants significantly increased some psychosocial constructs associated with SDT and MI and that there was a non-significant improvement in MVPA relative to usual care, providing further evidence of the potential effectiveness of a fully powered trial.

Randomized, controlled trials of physical activity interventions using SDT are appearing in the literature and show promise for increasing self-reported physical activity in the short term [34] and up to 2 years [26]. Silva et al. conducted a weight loss trial among 239 women and found improvements in exercise self-regulation, motivations for exercise, self-reported physical activity, and pedometer-assessed steps per day over 12 months in intervention compared with control participants [35]. Results persisted at 2 years [26]. Their trial was powered to be able to detect significant between-group differences, was twice as long as our pilot, and much more intense. Over the 12 months, about 60 h of intervention/participant contact time was scheduled compared with our average 2.5 h over 24 weeks [23]. However, Silva et al. conducted the intervention in groups of 25–30 women compared with our one-on-one approach. Although we did not find significant differences in MVPA, our impressive improvements in psychosocial variables representing SDT among intervention participants relative to usual care participants may suggest that fewer contact hours may be sufficient to obtain results using a one-to-one intervention, even if remotely delivered by telephone.

Even though the study was not powered to detect significant difference in biological outcomes, the 24-week intervention did not provide any signal of potential improvement. We learned that while most participants (82%) had visited their primary care provider within our follow-up window and, thus, had blood pressure and BMI measures in the EMR, only 34% had HbA1c or lipid values. We did not request providers to place laboratory orders for these outcomes, as it was beyond the scope of the pilot and we did not want to burden participants with the cost of a co-pay. In a trial with a longer intervention and follow-up period, more participants may have the assessments in their EMR. For those who do not, study staff can request that orders are placed and study funds can be used to cover the costs of co-pays. Physical activity interventions improve cardiometabolic risk factors [7], and it is important to continue to document these outcomes to convince primary care providers and health care systems to actively promote physical activity as a method to reduce risk.

Interviews with participants, providers, and clinic staff confirmed that the study is feasible. Participants were highly positive about all aspects, with the lone suggestion of the ability to meet with the interventionist at least once to improve the quality of interactions. Providers and clinic staff suggested that the study be more integrated into the clinic setting. While they did not want their workflows altered or be required to recruit patients into possible participation, they did believe that the study would benefit their patients. The time constraints in primary care offices are not trivial; feasible and sustainable approaches in this setting require limited involvement of providers and clinical staff [36–38]. The suggestions the providers and clinical staff provided to incorporate some of the study elements into existing clinical systems can be accomplished in a fully powered trial.

There are study limitations. Clinics were not randomly selected to be invited to participate in the pilot study because they were selected by convenience they may have been more responsive to the intervention. The interventionist only had 2 days of MI training and the calls were not assessed for fidelity. She may have strayed from the MI approach. Only four study participants were interviewed for the qualitative data collection and they all were assigned to the intervention arm. Qualitative results may have been different if all participants, both from the intervention and control arms, participated in interviews. Results of this study may not be generalizable to other health care settings. Additionally, given the exploratory nature of the study, results should be interpreted with caution.

In conclusion, the objectives of this pilot study were met. We demonstrated that the methods for identifying potential participants, data collection, and intervention were feasible and well-received by participants, providers, and clinic staff. Intervention participants exhibited improvements in SDT constructs, and there was a signal that accelerometry-derived MVPA improved relative to usual care participants. We learned about alternative methods to assess a potential participant’s medical ability to increase MVPA, given the busy clinic environment, and received useful advice for how to better incorporate study elements into primary care clinics. If a fully powered trial is successful, primary care settings with “behind-the-scenes” information technology support may be appropriate for increased MVPA among patients with prediabetes and diabetes, which would have a major impact on their health.

## Abbreviations

EMR: Electronic medical record
EVS: Exercise vital sign
HbA1c: Glycated hemoglobin
MI: Motivational interviewing
MVPA: Moderate to vigorous physical activity
NHANES: National Health and Nutrition Examination Survey
SDT: Self-determination theory
